# Mycelial frontiers: harnessing fungal morphological superiority and enzymatic versatility for deep-soil crude oil restoration in the Niger Delta

**DOI:** 10.3389/fmicb.2026.1809066

**Published:** 2026-05-18

**Authors:** Maryam Eyya Shuaib, Aman Thakur, Roberto Parra-Saldivar, Frederic Coulon, Angel Medina

**Affiliations:** 1 Magan Centre of Applied Mycology (MCAM), Cranfield University, Cranfield, United Kingdom; 2 Faculty of Engineering and Applied Sciences, Cranfield University, Cranfield, United Kingdom

**Keywords:** bioprospecting, crude oil, Lignin-Modifying Enzymes (LMEs), mycoremediation, Niger Delta

## Abstract

The extensive environmental degradation resulting from decades of crude oil contamination in the Niger Delta necessitates a critical re-evaluation of current restoration strategies. This crisis has persistently impacted soil and water resources, decimated mangrove forests, and undermined the socio-economic stability of local communities. An appraisal of current remediation efforts reveals a significant performance gap, with conventional techniques proving inadequate for the region's unique environmental conditions and the sheer depth of contamination. This review critically assesses the potential of mycoremediation in aerated environments as a superior, ecologically congruent alternative. We explore the distinct morphological and metabolic advantages of fungi over bacteria, particularly the ability of filamentous mycelial networks to penetrate and colonize deep soil matrices, thereby overcoming the critical depth limitations of existing methods. The paper investigates dual mechanisms of fungal action: potent enzymatic degradation via non-specific extracellular enzymes and non-enzymatic biosorption of pollutants. Furthermore, it analyzes how key environmental modulators in the Niger Delta, specifically acidic pH, mesophilic temperatures, and fluctuating water potential naturally select for fungal dominance. The review concludes by advocating for a paradigm shift away from generic microbial solutions toward bioprospecting and cultivating potent, indigenous fungal strains pre-adapted to local contaminants. Ultimately, this paper frames mycoremediation as a low-cost, decentralized technology capable of empowering communities, restoring livelihoods, and advancing environmental justice in this critically polluted region.

## 1 Introduction

**Figure 1:**
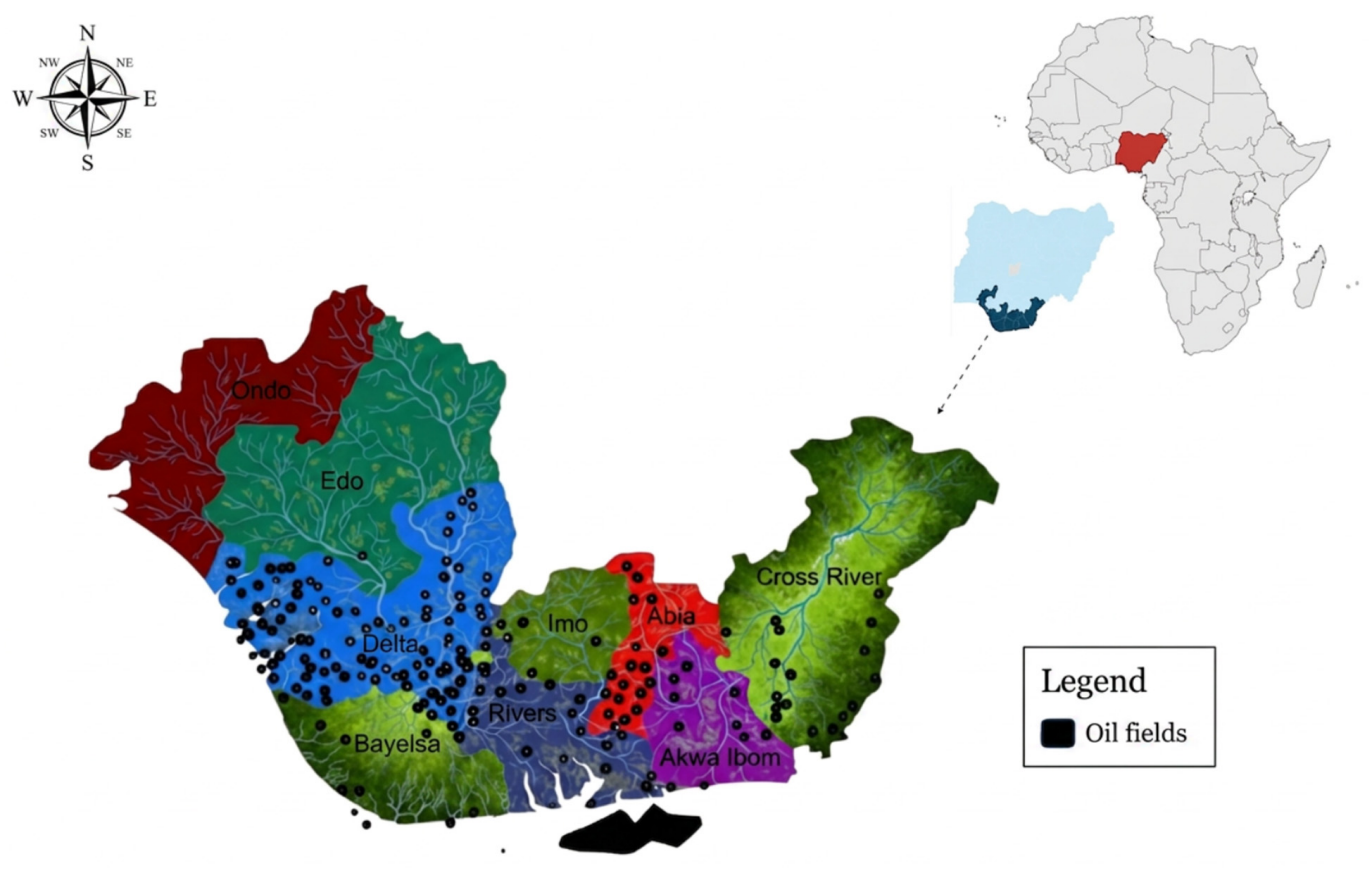
Map of the Niger Delta showing the extensive distribution of oil spill areas. The image has been adapted from Wekpe et al. (2024).

While crude oil remains the main energy resource driving global economic development and societal growth, its extraction and transport have resulted in persistent environmental contamination. This global pollution legacy specifically threatens the integrity of sensitive water and soil matrices, with disproportionate impacts on high-biodiversity coastal ecosystems, including saltmarshes, coral reefs, and mangrove forests. For decades, intensive and often poorly regulated oil exploitation has resulted in a legacy of severe environmental degradation, with chronic pollution affecting communities across the region (Sam et al., 2024). The scale of this contamination is exemplified by areas such as Ogoniland, which has been identified as one of the most severely polluted zones on the planet (UNEP, 2011). The geographic distribution of this contamination is vast, which maps the widespread nature of oil spill sites throughout the region (Figure 1).

The consequences of oil spillage pollution are multifaceted and with important social, economic and environmental consequences. The environmental burden is not static but continues to escalate, with aged contamination presenting a particularly formidable challenge. According to the Nigerian Upstream Petroleum Regulatory Commission (NUPRC), 732 oil spill incidents were reported in 2024, of which 59.01% were attributed to sabotage (NUPRC, 2024). Oil spillage can lead to fire breaks, deteriorate air and water quality, destroy non-renewable resources, and threaten the safety of seafood intended for human consumption (Okeke et al., 2022). The chemical composition of crude oil, particularly its content of toxic, mutagenic, and carcinogenic compounds like polycyclic aromatic hydrocarbons (PAHs), poses a direct toxicological risk (Okeke et al., 2022). PAHs are of particular concern due to their potential to damage DNA, and these lipophilic compounds can bioaccumulate in food chains, leading to long-term health issues (Okeke et al., 2022; Ławniczak et al., 2020). The ecotoxicological impacts of these spills are profound, threatening the entire ecosystem and human health through multiple pathways. The toxic aromatic hydrocarbons within crude oil pollute water bodies and infiltrate the food chain, posing a direct risk to human populations that rely on these resources (Okeke et al., 2022). The persistence of these pollutants in soil and sediment creates toxic “dead zones,” particularly damaging to mangrove forests, which are critical carbon sinks. The ecological devastation in the Niger Delta translates directly into a profound human crisis, systematically undermining the traditional livelihoods of local communities.

In recent years, various methods, such as physical, chemical, or a combination of both, have been used to address pollution from oil spills. These methods include excavation, incineration, landfarming, and the addition of chemicals like hydrogen peroxide. However, with chemical methods, the issues persist and could even worsen due to incomplete degradation (Shi et al., 2023), the production of harmful by-products, and the accumulation of greenhouse gases. For instance, the ozonation product 2-anilino-5-[(4-methylpentan-2-yl)amino]cyclohexa-2,5-diene-1,4-dione (6PPD-quinone), which is produced from the widely used tire rubber antioxidant *N*-(1,3-dimethylbutyl)-*N*-phenyl-*p*-phenylenediamine (6PPD), has the potential to harm marine life (Wang and Wang, 2021). Additionally, these processes are expensive, require high-maintenance equipment, and are not environmentally sustainable. In recognition of these problems with physical and chemical remediation techniques, current interests have adopted biological techniques over the past decades.

Bioremediation strategies have been adopted on a small and large scale to break down hydrocarbons *via in-situ* or *ex-situ* methods (Akpasi et al., 2023). Bioremediation techniques reported in the region include biostimulation and bioaugmentation, remediation by enhanced natural attenuation (RENA), and the use of biosurfactants. Biostimulation involves the introduction of nutrients to stimulate the activities of natural microorganisms at any stage of the treatment process. This includes methods like RENA, biocell, and Landfarming. RENA improves several environmental and chemical factors to optimize the activities of local microorganisms that break down hydrocarbons. This process involves regular tilling, diluting the pollutant with less contaminated soil, and adding fertilizers, enzymes, and biosurfactants. UNEP (2011), reported that RENA proved ineffective where contaminants are at depths beyond 1 m. Also, the contaminants have gone beyond 5 m deep as found on many sites in the Niger Delta area (Adesipo et al., 2020). Fungi have a remarkable capacity to metabolize diverse hazardous chemicals, which has prompted consideration for their application in bioremediation. Mushroom-forming fungi, primarily basidiomycetes, stand out as potent natural decomposers due to their vigorous growth and substantial biomass yield, they secrete robust extracellular enzymes. Notably, these enzymes encompass lignin peroxidases (LiP), manganese peroxidase (MnP) and laccase capable of degrading PAHs contaminations (Magan et al., 2022). The Niger Delta exhibits physical and microbiological attributes that are conducive to natural and biological remediation. Furthermore, the Niger delta is an area characterized by high rainfall and sufficient moisture, mesophilic temperatures (25–32 °C) which are suitable for fungal growth and proliferation of microorganisms.

In this review, we critically assess the viability of mycoremediation as a sustainable and socially equitable strategy for addressing crude oil contamination in the Niger Delta. We synthesize the mechanistic advantages of fungal-based systems over conventional remediation technologies and evaluate the ecological compatibility of native fungal species with the region's environmental conditions. Finally, we examine evidence from field applications and bioprospecting studies and propose a practical framework to support future community-driven implementation.

## 2 The case for mycoremediation in the Niger Delta: a critical analysis

### 2.1 The failure of current remediation paradigms in the Niger Delta

The Niger Delta region of Nigeria is a vast coastal region defined by a unique hydro-geography, spanning approximately 70,000 square kilometers and supporting a dense population of around 31 million people (UNPO, 2026). The region's soil, a complex matrix of sand and clay, combined with a perpetually humid climate, sustains one of the world's most critical wetland ecosystems (Azuazu et al., 2023). This environmental context is of global significance, as the Niger Delta contains the largest mangrove forest in Africa and is recognized for its exceptional biodiversity, leading the International Union for Conservation of Nature (IUCN) to designate it as a high conservation priority (Onyena and Sam, 2020). Among the species it harbors are *Avicennia* sp., *Rhizophora* sp., and *Laguncularia racemosa*, which play crucial roles in carbon storage, nutrient cycling, and shoreline protection (Aransiola et al., 2024). For the local inhabitants, these ecosystems are the foundation of socio-economic stability, providing essential resources for fuel, medicine, and artisanal fisheries, which are a primary source of protein and income (Barenblitt et al., 2024). This intimate connection between the people and their environment renders the population acutely vulnerable to ecological disruption, making the ongoing pollution crisis a direct threat to both a global ecological treasure and the communities it sustains (Ordinioha and Brisibe, 2013). The mangrove ecosystem of the Niger Delta provides indispensable goods and ecological services, forming the bedrock of the regional environment and economy. These services include shoreline protection, carbon sequestration, and the provision of habitats for numerous fish species, which in turn supports local livelihoods (Aransiola et al., 2024). The complex network of rivers, streams, and creeks flowing into the Atlantic Ocean creates a brackish water environment that hosts a rich biodiversity, including multiple species of mangrove trees, shellfish, and finfish (Aransiola et al., 2024). This natural infrastructure not only stabilizes the coastline against erosion but also enhances water quality and provides a variety of plant products used for medicine and fuel, highlighting its critical role in both environmental health and human wellbeing (Aransiola et al., 2024). This systemic failure, where the economic driver of the problem also inhibits its solution, demonstrates that the environmental crisis is not merely a technical challenge of pollution but a deeply entrenched socio-ecological catastrophe requiring a fundamentally new approach. This crisis is compounded by a significant governance failure, which has left communities with little recourse for the damage they have suffered. Under Nigerian law, oil companies are required to pay compensation unless spills are caused by sabotage, an argument frequently used to deny responsibility (Climate Diplomacy, 2026). The judiciary has often been slow and costly for communities to access, and there is a widespread lack of trust in both corporate and governmental oversight (Climate Diplomacy, 2026). This has forced affected populations, such as those in Bodo, to seek justice in international courts, highlighting the systemic impediments to achieving environmental justice within the national framework (Climate Diplomacy, 2026). The outcome is persistent environmental degradation driven by recurring oil spills and the weak enforcement of regulatory policies.

### 2.2 The case for mycoremediation: mechanisms and advantages

#### 2.2.1 Morphological superiority: mycelial networks as ecological engineers

One key reason for the effectiveness of fungi in soil remediation lies in their distinctive physical structure. Fungi possess a significant morphological advantage over bacteria for soil bioremediation, primarily due to their filamentous growth form, which directly addresses the critical “depth mismatch” plaguing conventional methods (Benguenab and Chibani, 2021). The fungal mycelium, a vast and interconnected network of hyphae, functions as an “ecological engineer” by actively exploring and penetrating the soil matrix far more efficiently than single-celled bacteria (Daccò et al., 2020). This invasive growth allows fungi to bridge air-filled gaps between soil particles and reach pockets of contamination deep within the soil profile, a capability that bacteria lack (Benguenab and Chibani, 2021; Covino et al., 2016). This unique physical attribute is a primary reason why fungi are exceptionally well-suited for remediating the complex, deep soil contamination characteristic of the Niger Delta.

This morphological superiority provides fungi with a distinct advantage in water-unsaturated environments where bacterial motility is severely restricted (Daccò et al., 2020). Unlike bacteria, which depend on continuous water-filled pathways for movement, filamentous fungi can translocate resources efficiently through their mycelial network, allowing them to grow across air-water interfaces and connect fragmented soil habitats (Daccò et al., 2020; Khanafer et al., 2025). This ability to navigate dry or partially saturated soil pores gives fungi unparalleled access to pollutants that are physically inaccessible to bacteria, making them more resilient and effective remediation agents in the fluctuating moisture conditions typical of the Niger Delta (Khanafer et al., 2025). However, fungi do not only rely on morphological structures, but there are also other factors such as enzymes which significantly play a central role in degradation of pollutants like crude oil and affects the outcome of remediation.

#### 2.2.2 Metabolic powerhouse: the non-specific enzymatic arsenal of fungi

In addition to their physical reach, fungi are metabolically adapted for the decomposition of highly recalcitrant organic matter, possessing a formidable arsenal of non-specific extracellular enzymes (da Rocha et al., 2023). As natural decomposers of durable biopolymers like lignin, fungi have evolved to secrete potent enzymes, such as peroxidases and laccases, that can fortuitously attack the structurally similar aromatic rings of pollutants like PAHs (da Rocha et al., 2023; Navina et al., 2024). This non-specific enzymatic machinery is a key advantage, as it allows fungi to degrade a wide range of complex organic contaminants without requiring prior adaptation or genetic modification (Navina et al., 2024). A valuable source of these enzymes is SMS, the residual material from mushroom cultivation, which can be repurposed as a low-cost, nutrient-rich amendment to enhance bioremediation (Gupta et al., 2024; Mayans et al., 2024). This combination of deep physical penetration and powerful, broad-spectrum biochemistry makes fungi ideal candidates for a new remediation paradigm. This enzymatic system includes both intracellular mechanisms, such as the cytochrome P450 system, and a suite of extracellular enzymes, with the latter being primarily responsible for breaking down large, complex pollutants (Daccò et al., 2020). The most critical of these are the Lignin-Modifying Enzymes (LMEs), which evolved to decompose lignin but exhibit low substrate specificity, allowing them to oxidize a wide array of structurally similar pollutants (da Rocha et al., 2023). This process is oxidative rather than hydrolytic, involving the withdrawal of electrons to destabilize and break down complex aromatic structures, a mechanism that is highly effective against the recalcitrant components of crude oil (da Rocha et al., 2023). Fungi employ both intracellular and extracellular enzymes to metabolize hydrocarbons, though the latter are primarily responsible for degrading the large, complex molecules found in crude oil (Sar et al., 2024). While intracellular systems like the cytochrome P450 monooxygenase (CYP) are linked to alkane metabolism, the primary strength of fungi lies in their secretion of a powerful suite of non-specific extracellular enzymes (Daccò et al., 2020). The most critical of these are the Lignin-Modifying Enzymes (LMEs), including laccases and peroxidases, which evolved to break down the highly durable biopolymer lignin (da Rocha et al., 2023). Because these enzymes exhibit low substrate specificity, they can fortuitously attack and oxidize a wide range of structurally similar pollutants like PAHs, initiating their breakdown into less complex and more bioavailable forms (AbuQamar et al., 2023). This non-specific oxidative power is the key biochemical mechanism that enables fungi to degrade the recalcitrant aromatic fractions of crude oil (da Rocha et al., 2023; Navina et al., 2024). The LME family includes several key enzymes, such as lignin peroxidase (LiP), manganese peroxidase (MnP), versatile peroxidase (VP), and laccases, each with distinct oxidative mechanisms (da Rocha et al., 2023). Peroxidases like LiP and MnP utilize hydrogen peroxide to generate highly reactive radicals that can attack and depolymerize complex aromatic structures. Laccases, which are multicopper oxidases, use molecular oxygen for oxidation and can act on a wide range of phenolic compounds (da Rocha et al., 2023; Silva Monteiro et al., 2024). The non-specific nature of these enzymes allows them to degrade a broad spectrum of xenobiotics, making them highly effective tools for breaking down the diverse mixture of compounds found in crude oil. Collectively, this versatile and non-specific enzymatic system enables fungi to efficiently transform and mineralize a wide spectrum of recalcitrant hydrocarbons, reinforcing their central role in effective mycoremediation strategies.

#### 2.2.3 Non-enzymatic mechanisms: biosorption and bioavailability enhancement

Although numerous studies have reported the efficacy of bacterial biosorption (Sanches et al., 2017), the process is mechanistically complex and involves more than simple physicochemical adsorption (Xu et al., 2013). Bacteria play an active role in waste sequestration through metabolism-dependent bioaccumulation and the secretion of extracellular polymeric substances (EPS) and enzymes that facilitate the capture and transformation of contaminants (Sun et al., 2026; Ali Redha, 2020; De Santis et al., 2026). However, fungal biosorption offers distinct advantages in complex environmental matrices such as soil. Unlike unicellular bacteria, the filamentous growth of fungal hyphae provides a superior surface area to quantity ratio, allowing for deeper penetration and more extensive physical contact with contaminants (Dinakarkumar et al., 2024). Furthermore, while bacterial activity can be sensitive to fluctuating pH and nutrient scarcity, many fungi, particularly white-rot species, maintain high biosorption capacities and metabolic activity under acidic or nutrient-poor conditions, often outperforming bacterial systems in the remediation of recalcitrant pollutants (Sanches et al., 2017). This physicochemical process involves the passive adsorption of pollutants onto the surface of the fungal mycelium, which is rich in functional groups capable of binding with contaminants through mechanisms such as complexation and adhesion (Dinakarkumar et al., 2024). This process works in powerful synergy with enzymatic degradation by overcoming the critical limitations of pollutant bioavailability (Daccò et al., 2020). The vast surface area of the mycelial network first acts as a passive filter to trap and concentrate hydrophobic hydrocarbons, after which the secreted extracellular enzymes can attack the concentrated substrate with maximum efficiency, creating a highly effective “one-two punch” of capture and degradation. This integrated system is far more efficient than relying on random molecular encounters in a heterogeneous soil matrix. Biosorption is a critical first step that addresses the challenge of bioavailability, which is a major limiting factor in the remediation of aged, soil-bound hydrocarbons (Daccò et al., 2020). This process can be either metabolism-dependent (bioaccumulation) or metabolism-independent (mycosorption), with the latter relying on the physicochemical properties of the fungal cell wall (Dinakarkumar et al., 2024). Functional groups such as carboxyl, hydroxyl, and amine on the mycelial surface attract and bind pollutants, effectively concentrating them for subsequent enzymatic attack (Dinakarkumar et al., 2024). This mechanism is particularly important for hydrophobic compounds that are otherwise poorly accessible to microbial action, demonstrating a key advantage of mycoremediation over purely bacterial approaches (Gupta et al., 2024). Additionally, many fungal species produce biosurfactants, such as lipopeptides, which reduce surface tension and emulsify hydrocarbons (Sonbhadra and Pandey, 2025), further increasing their availability for microbial uptake. This integrated combination of mechanisms highlights the distinct advantage of fungi in overcoming bioavailability limitations, thereby enabling more efficient and sustained remediation of hydrocarbon-contaminated soils.

#### 2.2.4 The fungal-bacterial consortium: mycelial networks as microbial highways

The fungal advantage extends beyond their individual capabilities to fostering powerful synergistic relationships with bacteria, enhancing the overall efficacy of bioremediation (Song et al., 2022). Fungal hyphae can serve as vectorial transport providing continuous surfaces and channels that facilitate the transport of pollutant-degrading bacteria into deeper soil regions that they could not otherwise access (Song et al., 2022; Ossai et al., 2022). This interaction reframes the remediation model from a competition between microbial domains to a powerful consortium to function more effectively (Wei et al., 2024; Atakpa et al., 2022). Such synergistic cooperation is crucial for the complete mineralization of complex hydrocarbon mixtures found in crude oil.

Beyond their physical role in microbial transport, fungal mycelial networks also contribute chemically to pollutant immobilization through the functional properties of the fungal cell wall. Cell wall components such as chitin, glucans, and associated proteins which could provide functional groups capable of binding heavy metals and organic pollutants. Under acidic conditions, partial deacetylation of chitin to chitosan increases the availability of hydroxyl and amino groups, enhancing contaminant biosorption onto fungal hyphae. This process concentrates pollutants along the mycelial network, creating microenvironments that promote subsequent microbial degradation (Geetha et al., 2021). In turn, bacteria can assist in hyphal growth and protect fungi from parasites (Wei et al., 2024). This co-metabolism, where fungi initiate the breakdown of complex molecules and bacteria mineralize the simpler intermediates, creates a more robust and efficient degradation process, making fungal-bacterial consortia a highly promising strategy for bioremediation (Silva Monteiro et al., 2024; Atakpa et al., 2022). Studies have shown that mixed consortia can achieve significantly higher degradation of TPH and recalcitrant PAHs than pure cultures, with some reporting removal rates exceeding 93% (Silva Monteiro et al., 2024). However, these interactions are not universally synergistic. In soil environments, competition for nutrients, oxygen, and space, as well as antagonistic metabolite production, may suppress key degraders and reduce remediation efficiency. Community instability under fluctuating environmental conditions can further disrupt cooperative networks. Therefore, while fungal–bacterial consortia are highly promising, their performance depends on ecological compatibility and environmental optimisation.

#### 2.2.5 A comparative analysis of microbial degradation efficiencies

The biodegradability of hydrocarbons generally follows a hierarchy based on molecular complexity, with simpler molecules like linear alkanes being degraded more readily than complex, high-molecular-weight compounds such as asphaltenes. Within this framework, quantitative evidence demonstrates that fungi are highly effective agents for hydrocarbon removal, with degradation efficiencies often comparable or superior to their bacterial counterparts (Daccò et al., 2020). Studies have consistently shown that different fungal species exhibit varying capabilities for degrading specific hydrocarbon fractions. one study found that *Aspergillus niger* achieved 58% biodegradation of crude oil, outperforming *Aspergillus polyporicola* (47%) and *Aspergillus spelaeus* (51%) (Al-Dhabaan, 2021). Another study demonstrated that while *Aspergillus oryzae* could degrade all hydrocarbon components in used engine oil, *Mucor irregularis* was only effective against long-chain hydrocarbons and BTEX compounds (Asemoloye et al., 2017). This specificity highlights the importance of selecting the appropriate fungal strains or, more effectively, using a consortium of different species to ensure the comprehensive degradation of the complex mixture of pollutants found in crude oil spills. Table 1 summarizes reported degradation efficiencies for various microbial genera against different hydrocarbon classes. Fungal species, such as *Penicillium javanicum* and *Purpureocillium lilacinum*, demonstrate high efficacy (>90%) for alkane degradation. This quantitative comparison supports the strong potential of mycoremediation relative to other microbial strategies for hydrocarbon cleanup.

**Table 1:** Hydrocarbon degradation efficiencies of selected microorganisms under varying experimental conditions.

Microorganisms	Hydrocarbons degradation efficiency (%) ^*^	Degradation medium	Employed method	Experimental conditions	References
*Yarowia lipolytica, Candida bombicola*	n-Hexadecane (75%)	SC medium (without glucose) supplemented with *n*-hexadecane (10 g/L or 20 g/L)	Batch degradation experiment	30 °C, 220 rpm, 96 h	Shang et al. (2024)
*Candida*	Complete metabolize of n-alkanes, Anthracene (51%), Fluoranthene (74%)	Mineral medium (MM) supplemented with hydrocarbons (e.g., *n*-alkanes, petroleum compounds, 1% hydrocarbons as sole carbon source); working volume: 50 mL	Batch biodegradation assays	30 °C, 180 rpm; incubation period up to 12 days; inoculum ~10% (v/v)	Gargouri et al. (2015)
*Trichosporon*	Phenanthrene (47%), Anthracene (73%), Fluoranthene (31.4%)				
*Candida digboiensis*	Total Petroleum Hydrocarbons (73%)	Minimal salts medium (MSM, pH 3) supplemented with petroleum hydrocarbons/oily sludge (0.1–1% w/v) as sole carbon source; working volume: 50 mL	Batch biodegradation assays	30 °C, 180 rpm; incubation period ~7 days; inoculum 5% (v/v	Sood and Lal (2009)
*Alternaria tenuissima 5c-12 and Epicoccum nigrum 3b-1*	Long-chain hydrocarbons including C8-C16 alkanes, C36 n-alkane and Pristane (70%)	Bushnell–Haas medium supplemented with petroleum (1%) or PAHs (0.2 g/L total), DCPIP (2%), Tween 80 (0.1%); working volume: 50 mL	Batch biodegradation assays	26 °C, 180 rpm; incubation up to 47 days (liquid assay); soil assay up to 60 days with 10% (v/w) petroleum and inoculum	Fallahi et al. (2023)
*Penicillium javanicum*	Pristane (>95%)	Mineral salt medium (pH 5.4) supplemented with substrates (as sole carbon source); working volume ~100 mL	Batch biotransformation assays	30 °C, 130 rpm (shaking) or static; incubation period 7 days	Gaid et al. (2023)
*Purpureocillium lilacinum*	Pristane (>90%)				
*Mucor circinelloides*	Aliphatic hydrocarbons in 25 g L^−1^ NaCl (87%)	Mineral salts medium (MSM) supplemented with crude oil (10 g/L or 50 g/L) as sole carbon source, with NaCl (0–100 g/L); soil microcosm with 10–50 g/kg crude oil	Batch biodegradation assays	28 °C, 170 rpm; incubation up to 12 days (liquid) and 105 days (soil); inoculum ~5 × 105 spores/mL (liquid) or 5% spore suspension (soil)	Azin et al. (2022)
*Embellisia sp. KJ59 and Alternaria sp. KJ66*	Petroleum (81.3 to 95.1%) and Pyrene (>88.3%)	Minimal salt medium (MSM) or Potato Dextrose Broth (PDB) supplemented with crude oil (1% v/v) or pyrene (100 mg/L) as sole carbon source, with NaCl (0–5% w/v); Working volume: 10 mL	Batch biodegradation assays	28 °C, 170 rpm; incubation up to 14 days; inoculum ~5 × 10^6^ spores/mL (5% v/v)	Jenab et al. (2023)
*Burkholderia* sp.	Total petroleum hydrocarbons (46.7%)	Petroleum-contaminated soil (28,360 mg/kg TPH) containing macro alkanes (C16–C30); nutrient amendments (glucose, acetic acid, trace elements); pre-oxidation with H_2_O_2_ and novel iron	Combined pre-oxidation (Fenton-like process) and bio-stimulation	Pre-oxidation: 450 mM H_2_O_2_, ~2 g iron, 24 h, pH adjusted to 6.5–7.5; Bio-stimulation: up to 80 days, intermittent shaking at 200 rpm, periodic aeration	Xu et al. (2023)
*Pseudomonas aeruginosa*	Short-chain (C5–C10) (91.9%), Long-chain (C10–C40) (86%)	Nutrients and trace elements, glucose, meat extract; petroleum diesel added (5–10 mL) for enrichment	Pre-test screening in 3 L artisanal bioreactors with constant aeration (3 L/min); biotreatment in 1 L reactors in triplicate for 7 days	Temperature: 37 °C; aeration.	Cabello-Torres et al. (2023)
*Actinobacteria*	Increased the removal from 55.3% (C5–C10) to 65% (C10–C40)				

^*^Degradation efficiencies are not directly comparable across microorganisms due to differences in hydrocarbon substrates, initial concentrations, incubation periods, and experimental conditions reported in the respective studies.

## 3 Ecological congruence: why mycoremediation is inherently suited to the Niger Delta?

### 3.1 Soil chemistry: advantage in acidic pH and high salinity

An additional advantage of fungi lies in their strong alignment with the native physicochemical conditions of the Niger Delta. The specific environmental profile of the Niger Delta creates conditions that naturally select for the dominance of fungi, a pattern that can be described as “ecological congruence.” The region's predominantly acidic soils, with a typical pH range of 5.7 to 5.9, confer a significant competitive advantage to fungi, which are generally more tolerant of acidic conditions than many bacteria (Benguenab and Chibani, 2021; Dinakarkumar et al., 2024). In fact, fungi can contribute to further acidification through the secretion of organic acids, reinforcing their dominance in this environment (Daccò et al., 2020). Furthermore, fungi demonstrate superior resilience to the high salinity found in the coastal and mangrove zones of the delta; by accumulating internal compatible solutes, fungi can counteract the significant osmotic stress imposed by high external salt concentrations, a physiological mechanism that is less common in many bacterial species (Fontana et al., 2024). This inherent tolerance for both acidic and saline conditions make fungi robust and pre-adapted candidates for remediation in the native soils of the region (Dinakarkumar et al., 2024). These attributes demonstrate that fungi are not only well-suited but inherently pre-adapted to the prevailing environmental conditions of the Niger Delta, reinforcing their effectiveness as reliable agents for *in situ* bioremediation.

This competitive advantage is further reinforced by the broad physiological resilience of fungi to multiple environmental stresses commonly associated with contaminated sites. Fungi are known to have a higher potential to survive harsh environmental conditions, such as extreme pH and poor nutrient status, compared to other microorganisms (Dinakarkumar et al., 2024). This adaptability makes them particularly well-suited for contaminated sites where conditions may be unfavorable for bacterial growth (Dinakarkumar et al., 2024). Studies on fungi isolated from extreme environments, such as hydrothermal vents, confirm their capacity to withstand high salinity and degrade complex hydrocarbons, suggesting that similar adaptations exist in fungi from the saline, hydrocarbon-impacted environments of the Niger Delta (Stanley et al., 2022). Research on *Pleurotus ostreatus* has shown that its mycelium can tolerate estuarine salinities, with maximum growth observed between 5 and 15%, conditions that are representative of the delta's brackish waters (Covino et al., 2016). Together, this ecological congruence and physiological resilience highlight the strong potential of fungi as naturally adapted, efficient, and sustainable agents for bioremediation in the complex environmental conditions of the Niger Delta.

### 3.2 Thermal profile: dominance in mesophilic conditions

The efficacy of microbial degradation is strongly influenced by temperature, which affects metabolic rates and enzyme activity (Asemoloye et al., 2017). The Niger Delta's warm, tropical climate provides mesophilic conditions year-round, which aligns with the optimal temperature range for the growth and enzymatic activity of many potent hydrocarbon-degrading fungi (Asemoloye et al., 2017; Ahmad et al., 2024). This constant warmth favors sustained fungal activity, avoiding the significant slowdown in biodegradation rates that occurs at lower temperatures. This thermal profile ensures that mycoremediation processes can operate at high efficiency throughout the year, a distinct advantage over regions with temperate climates where remediation may be seasonally limited.

The consistently warm temperatures in the tropics are known to enhance microbial activity and, consequently, bioremediation rates (Ahmad et al., 2024). Studies have shown that the degradation of organic contaminants is significantly higher in tropical soils compared to those from temperate regions, as temperature affects the half-life of pollutants (Asemoloye et al., 2017). The optimal temperature range for mycoremediation is generally between 25 and 30 °C, which aligns perfectly with the climate of the Niger Delta (Asemoloye et al., 2017). This thermal advantage suggests that mycoremediation approaches will be particularly effective and can be sustained year-round without the need for costly heating or insulation measures that might be required in cooler climates (Ahmad et al., 2024). Overall, the favorable thermal regime of the Niger Delta provides an inherent and sustained advantage for fungal activity, thereby enhancing the efficiency, consistency, and cost-effectiveness of mycoremediation processes in the region.

### 3.3 Water potential: resilience to fluctuating moisture

**Figure 2:**
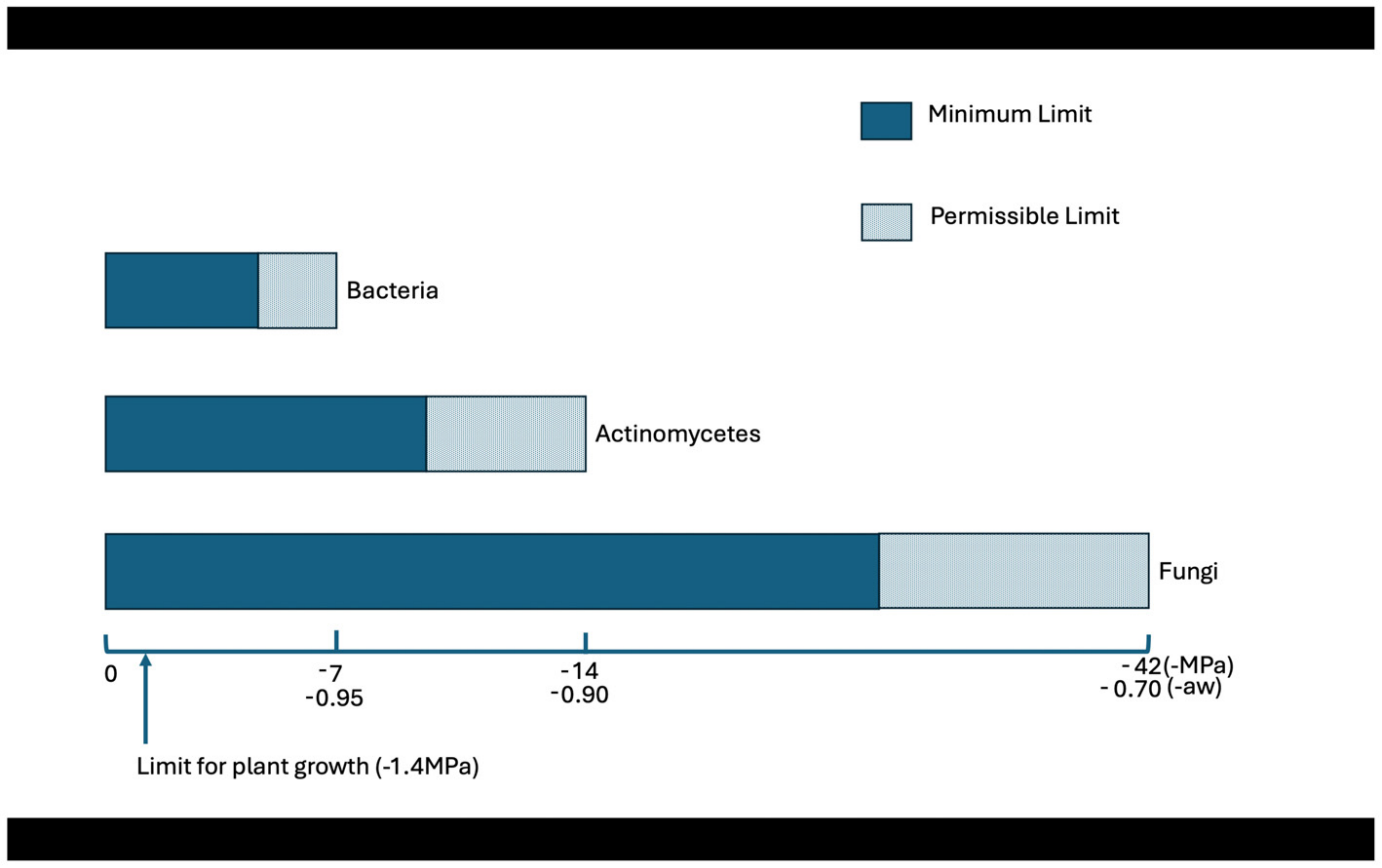
Conceptual diagram illustrating the distinct ecological niches of different microbial groups based on water availability. This figure was adapted from Magan (2007). The shaded areas represent relative dominance zones based on microbial tolerance to water potential rather than strict growth boundaries. The X-axis show soil water potential (Ψ) expressed in megapascals (MPa), which describe the energy status of water in soil systems.

Water availability, or water potential, is a critical master variable governing microbial activity and competition in soil ecosystems (Khanafer et al., 2025). Fungi can remain metabolically active at much lower water potentials, that is, in much drier conditions than most bacteria, a crucial advantage in environments with fluctuating moisture levels (Daccò et al., 2020). This resilience stems from their filamentous growth form; unlike bacteria, which rely on continuous water-filled pathways for movement, fungal hyphal networks can grow across air-filled pores, allowing them to continue exploring the soil matrix and accessing isolated pockets of water and nutrients as the soil dries (Daccò et al., 2020; Covino et al., 2016). Despite its importance, the differential effects of matric potential (water adhesion to soil particles) vs. solute potential (osmotic stress from dissolved salts) on mycoremediation efficacy remain a significant and under-researched knowledge gap critical for optimizing field applications. Figure 2 shows the ranges of water potentials/water activities over which different groups of microorganisms can effectively grow; bacterial activity predominates under relatively wet soil conditions where continuous water films facilitate nutrient diffusion and motility. In contrast, filamentous fungi and actinomycetes tolerate much lower water potentials due to their hyphal growth and osmotic stress tolerance, allowing them to remain metabolically active in drier soils. This is important if an individual or mixed inoculum will be introduced into the soil for remedial purposes. Without water films between the soil particles, bacteria are relatively inactive. In contrast, mycelial actinomycetes and filamentous fungi can grow via hyphal extension and colonize more expansive areas of the soil, enhancing the opportunity for degrading the xenobiotic compounds. Surprisingly, very few studies have examined the effect of matric vs. solute potential on the remediation of xenobiotic compounds such as crude oil. In addition, interactions with temperature and pH have received very little attention, if any. Furthermore, the combined effects of water potential, temperature, and pH remain poorly understood, constraining the translation of laboratory findings to field-scale applications.

The water potential relations of microbes in soil are essential. Under relatively wet conditions, bacterial activity is favored, while under drier conditions close to the wilting point of plants or much direr conditions, filamentous fungi and stress-tolerant actinomycetes activity is predominant.

**Figure 3:**
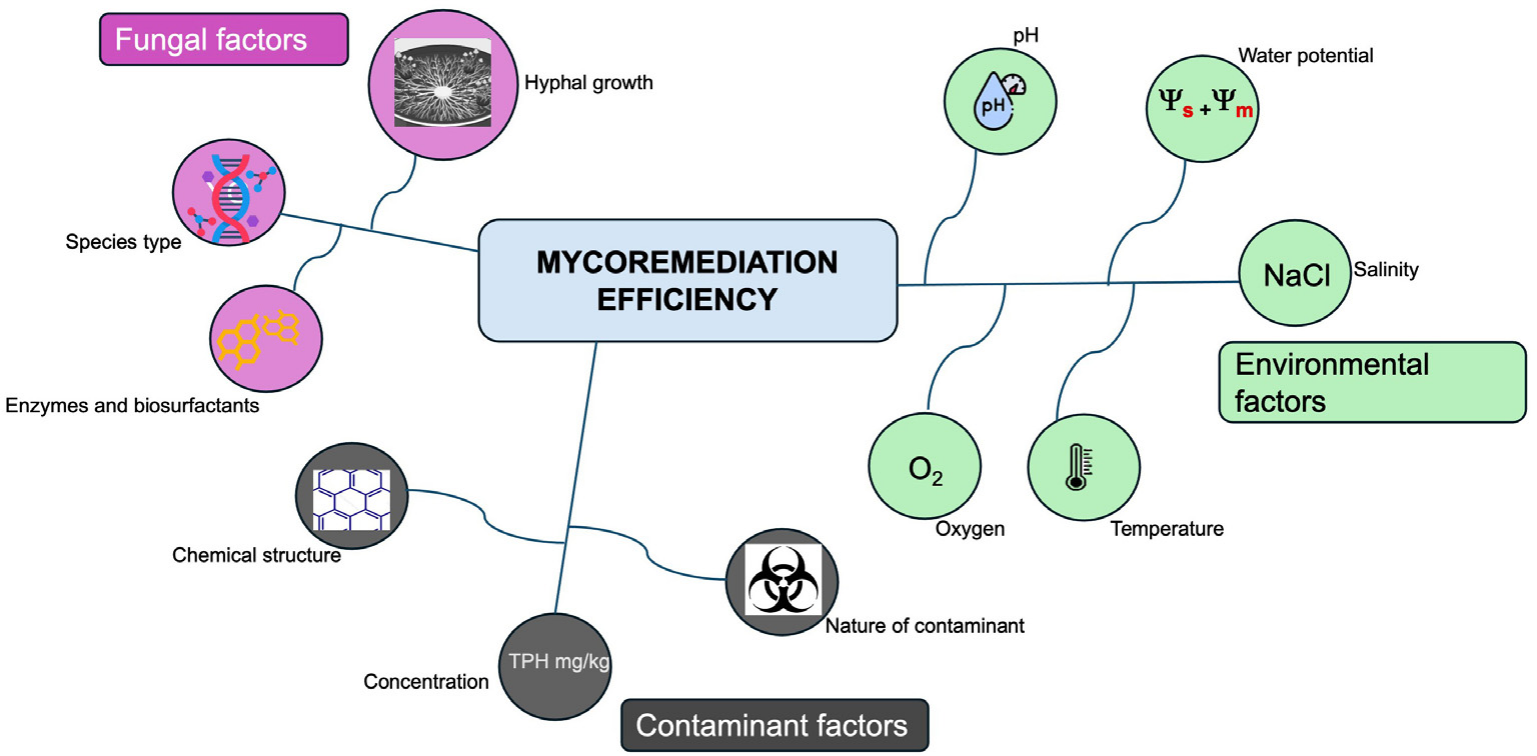
Schematic representation of the factors influencing mycoremediation efficiency. The image represents various fungal, contaminant and environmental factors that govern the success of mycoremediation. The interaction among these factors collectively determines fungal growth, pollutant accessibility, and the overall efficiency of the mycoremediation process.

The ability of fungi to thrive under varying moisture conditions is a key advantage for bioremediation (Dinakarkumar et al., 2024). The efficiency of mycoremediation is influenced by a range of environmental factors, including humidity, oxygen, and temperature (Sar et al., 2024). Figure 3 shows how fungal factors, environmental and contaminant factors drive Mycoremediation efficiency, hydrocarbon degradation is strongly influenced by environmental variables including temperature, oxygen availability, nutrient concentration, and water potential. These factors regulate microbial metabolic activity and therefore determine the efficiency of bioremediation processes, and this can vary between fungal species. Table 2 shows the key environmental parameters in the Niger Delta that influence microbial tolerance and hydrocarbon degradation. Environmental parameters such as pH, temperature, salinity, water availability, and oxygen levels collectively shape the relative dominance of fungi and bacteria, with fungi generally exhibiting higher tolerance to acidic and saline conditions.

**Table 2:** Fungal vs. bacterial responses to key environmental factors in the Niger Delta.

Factor	Typical range	Fungal preference/tolerance	Comparative microbial tolerance (fungi vs. bacteria)	Impact on hydrocarbon degradation
pH 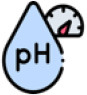	5.7–5.9 (acidic, shifts to neutral with oil) (Korneykova et al., 2021)	Higher tolerance to acidic conditions of pH < 4.	Fungi generally more tolerant to acidic pH than bacteria (Jumbo et al., 2022)	Influences enzyme activity and nutrient availability
Temperature 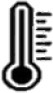	28 °C average, +2 °C during dry season	Mesophilic (optimal 25–40 °C)	Mesophilic bacteria/actinomycetes thrive 15–25 °C (Gross and Robbins, 2000) Filamentous fungi minimum, optimal, and maximum temperatures at approximately 5, 25, and 40 °C, respectively (Hesnawi and Mogadami, 2013)	Increases degradation rate; reduces oil viscosity
Salinity 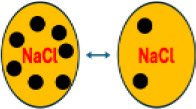	High salinity in coastal/estuarine areas	Resist higher osmotic pressure via polyol production	Fungi generally more tolerant than bacteria/actinomycetes (Gouma et al., 2014)	Can inhibit microbial activity; certain fungi thrive
Water Availability ψsolute +ψ matric	High rainfall, varying soil types	Predominant in drier conditions via hyphal extension	Bacteria favored in wet conditions; fungi/actinomycetes in drier (Magan, 2007)	Essential for microbial activity; influences pollutant access
**Oxygen** 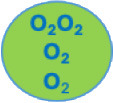	Aerobic conditions common, but can vary	Varying requirements: some tolerate low O_2_ (less than 10%)	Aerobic degradation generally faster (Azin et al., 2022)	Crucial for oxidative reactions

## 4 Bioprospecting for potent indigenous fungi: a targeted approach

### 4.1 Key fungal taxa in hydrocarbon degradation

The field of mycoremediation gained significant momentum with the landmark discovery that Basidiomycota, particularly “white-rot fungi” like *Phanerochaete chrysosporium*, could degrade highly recalcitrant molecules previously considered non-biodegradable (Ali et al., 2017). The phylum *Ascomycota* also contains numerous species with powerful bioremediation capabilities that are naturally present in the Niger Delta; genera such as *Penicillium, Trichoderma*, and *Aspergillus* are known to effectively degrade a wide range of PAHs and have been successfully isolated from contaminated sites in the region (Aranda, 2016; Ezekoye et al., 2018). The confirmed presence of these pre-adapted local strains provides a rich and highly relevant resource for developing targeted mycoremediation strategies.

A wide diversity of fungal genera has been identified for their ability to decontaminate petroleum hydrocarbons, including *Rhizopus, Candida, Syncephalastrum, Paecilomyces, Mucor, Yarrowia, Fusarium, Amorphoteca, Pichia, Graphium, Neosartorya*, and *Talaromyces* (Daccò et al., 2020; Song et al., 2022, 2024). *In-situ* studies of bioremediation sites in the Niger Delta have revealed a succession of fungal communities, with genera such as *Candida, Geotrichum, Sepedonium, Aspergillus, Phoma, Cladosporium, Scopulariopsis, Bipolaris, Gliocladium, Paecilomyces*, and *Trichophyton* being prominent (Ezekoye et al., 2018). This diverse pool of indigenous fungi represents a valuable genetic resource for developing locally adapted and highly effective mycoremediation solutions.

### 4.2 Evidence from the field: successful trials with native fungi

Recent field trials in the Niger Delta have confirmed the remarkable effectiveness of indigenous fungi, providing strong evidence that a bioprospecting approach is superior to introducing foreign organisms (Dickson et al., 2019). One study demonstrated that the application of local mushroom species, including *Pleurotus pulmonarius*, could achieve significant pollutant degradation in contaminated soil (Njoku et al., 2017). An even more striking result was observed in a recent field trial in Ogoniland that used the native fungus *Pleurotus ostreatus* and fermented palm wine, achieving over 98% TPH remediation across various local soil types (Dickson et al., 2019). These successful, locally conducted trials validate the high potential of fungi already adapted to the region's specific contaminants and environmental conditions.

A study using *Pleurotus pulmonarius* in Nigeria demonstrated significant TPH degradation, with removal percentages of up to 52.6% in soil with moderate contamination levels (Njoku et al., 2017). The same study also found a significant reduction in the concentration of heavy metals, including manganese, copper, and zinc, after 62 days of incubation (Njoku et al., 2017). These findings not only confirm the efficacy of locally sourced fungi but also highlight their potential for addressing the co-contamination of hydrocarbons and heavy metals, which is a common problem in the Niger Delta.

### 4.3 The case for locally adapted strains

The success of native fungi in field trials supports a paradigm shift away from implanting generic, lab-cultured microbial solutions toward a strategy of bioprospecting (Benguenab and Chibani, 2021). Isolating cultivable fungi from oil-polluted soils provides strains that are more adapted to the polluted conditions and more capable of competing with the native microbiome than strains obtained from culture collections (Benguenab and Chibani, 2021). The principle of natural selection dictates that in a chronically polluted environment, organisms with traits that allow them to tolerate or metabolize the pollutant will have a competitive advantage. The isolation of potent native degraders like *Aspergillus polyporicola* and *Purpureocillium lilacinum* from contaminated sites confirms their adaptation to local conditions (Benguenab and Chibani, 2021; Al-Dhabaan, 2021). Therefore, the most logical and ecologically sound strategy is to identify indigenous fungi that can accelerate the natural remediation process. The rationale for using indigenous strains is further supported by studies showing that fungi isolated from contaminated sites are more effective degraders than those from pristine environments (Benguenab and Chibani, 2021). The chronic exposure to hydrocarbons in polluted soils selects for fungal populations with enhanced metabolic capabilities for these compounds (Benguenab and Chibani, 2021). For example, studies in Nigeria have successfully isolated and identified potent hydrocarbon-degrading fungi such as *Aspergillus oryzae* and *Mucor irregularis* directly from contaminated fields, confirming their adaptation and efficacy (Asemoloye et al., 2017). Focusing on these pre-adapted, native fungi avoids the ecological risks associated with introducing non-native species and leverages the power of natural selection to find the most effective agents for bioremediation.

## 5 A framework for future research and sustainable implementation

### 5.1 Key research imperatives

To translate the promise of mycoremediation into a robust, field-ready technology for the Niger Delta, a targeted research agenda is required. A primary imperative is the development of synergistic fungal-bacterial consortia specifically tailored to the region's contaminants and environmental conditions, moving beyond single-organism approaches to harness the power of microbial teamwork (Wei et al., 2024; Atakpa et al., 2022). Concurrently, research must address the critical knowledge gap concerning the interactive effects of matric and solute water potential on remediation efficacy, as understanding this master variable is key to optimizing performance in a fluctuating climate. Finally, a shift in monitoring is needed, integrating advanced tools such as metagenomic sequencing to track microbial community dynamics and functional gene expression, rather than relying solely on the decline of TPH (Khanafer et al., 2025; Song et al., 2022). Recent research has underscored the superior performance of mixed microbial communities over pure cultures for degrading complex hydrocarbon mixtures (Wei et al., 2024). Fungal-bacterial consortia have demonstrated significantly higher degradation capacity for both TPH and recalcitrant PAHs, with some studies reporting over 93% removal (Silva Monteiro et al., 2024). Future research should focus on identifying and constructing optimal consortia from indigenous Niger Delta microbes (Khanafer et al., 2025; Atakpa et al., 2022), exploring the synergistic mechanisms at play, and using advanced tools like metagenomics to understand community dynamics and optimize performance for field applications.

### 5.2 Implementation strategy for community-based mycoremediation

A practical pathway for implementation should follow a phased approach, beginning with comprehensive bioprospecting to identify the most potent indigenous fungal strains from contaminated sites across the Niger Delta (Giwa et al., 2023). This discovery phase should be followed by lab-scale optimization of cultivation and application techniques, pilot-scale field trials to validate efficacy under real-world conditions, and finally, full-scale deployment (Giwa et al., 2023). Critically, this framework should be built on a decentralized, community-based model. Mycoremediation is a low-cost, low-technology approach that can utilize locally available substrates, making it exceptionally well-suited to empower local communities to take ownership of the cleanup process, which can foster local enterprise and ensure the long-term sustainability of restoration efforts (Giwa et al., 2023). A key element of this strategy is the use of low-cost, locally available organic materials as substrates and amendments. Spent Mushroom Substrate (SMS), the residual material from mushroom cultivation, is a prime example of a valuable resource that can be repurposed for bioremediation (Gupta et al., 2024; Mayans et al., 2024). SMS is rich in nutrients, fungal mycelia, and extracellular enzymes, making it an effective biofertilizer and remediation agent (Mayans et al., 2024). Similarly, other agricultural wastes, such as micronized keratinous wastes (human hair and chicken feathers), have been shown to act as effective biostimulants and bio-carriers, achieving over 90% TPH removal in polluted soil (Ossai et al., 2022). Integrating these waste-valorisation strategies into a community-based model can create a circular economy, turning agricultural waste into a tool for environmental restoration.

### 5.3 Mycoremediation

Ultimately, mycoremediation should be framed not only as a technically robust solution but as a transformative technology with the potential to advance environmental justice and restore livelihoods in the Niger Delta (Ujuagu et al., 2025). The decades of pollution have destroyed the traditional subsistence economies of farming and fishing, creating a cycle of poverty and disenfranchisement (Ordinioha and Brisibe, 2013). By offering a low-cost, accessible, and effective means to restore contaminated lands and waters, mycoremediation provides a direct pathway to reviving these local economies. Adopting this decentralized technology represents an opportunity to break the “paradox of plenty” and empower communities, positioning it as a powerful tool for achieving both ecological restoration and social equity in one of the world's most critically polluted regions.

The industrial pollution in Africa has imposed severe health burdens and socio-economic consequences on vulnerable populations, perpetuating cycles of poverty and environmental degradation (Ujuagu et al., 2025). In the Niger Delta, this has manifested as a loss of livelihoods, forced migration, and social unrest fueled by the destruction of natural resources (Ujuagu et al., 2025). By providing a tool that local communities can implement themselves, mycoremediation offers a pathway to reclaim environmental sovereignty. Empowering communities to restore their own land and water resources can help rebuild local economies, improve food security, and address the deep-seated social injustices that have characterized the region's history with the oil industry.

### 5.4 Current challenges

The challenges of biological remediation strategies have been highlighted by (Azuazu et al., 2023), where the drawbacks and management of biological techniques were effectively explained. One of the drawbacks of bioaugmentation methods is the lack of soil tests to evaluate the availability of hydrocarbon-utilizing microbes. The suggested mitigation was to use a combination of indigenous microbes singly or in combination in soils to enhance degradation (Chen et al., 2020). Nevertheless, a potential solution to this issue could be utilizing microbial strains extracted directly from crude oil. This approach represents a novel and exploratory avenue, as current literature lacks studies explicitly focused on bioremediation through such microbial isolates. The effectiveness of biostimulation techniques faces hurdles, including the improper or excessive application of fertilizers, leading to disproportionate nutrient availability for microbes, the challenge of securing sufficient quantities of fertilizers due to their high costs, and the influence of uncontrollable environmental variables. These challenges could be addressed by acknowledging that the ideal carbon:nitrogen:phosphorus (C:N:P) ratio of 100:10:1 might not universally apply in extensive remediation efforts. This is due to the variability in site-specific factors like soil characteristics, contaminant makeup, and pre-existing microbial populations. Both overapplication and underapplication of nutrients can impede the biodegradation process (Coulon et al., 2012). Nutrient limitation assessment should be carried out to supplement the specific nutrient needed in the right quantity/ratio to save time, resources, and cost. The challenges in implementing bioremediation in the Niger Delta exist even with the implementation of 2 major remediation projects. The first initiative, commissioned by the Nigerian Government in response to the UNEP report, involves Hydrocarbon Pollution Remediation Project (HYPREP) this was set up by the federal government of Nigeria to restore and revitalize communities impacted by hydrocarbon pollution, with a primary focus on the Ogoniland region. This extensive effort is projected to span 20 to 30 years to achieve complete ecological restoration. The second environmental remediation endeavor is the Bodo Creek project, spearheaded by the Shell Petroleum Development Company of Nigeria (SPDC). This project stems from an out-of-court settlement in the Bodo Vs SPDC case of 2013 projects have opted for a bioremediation strategy, as evidenced by studies conducted by Gbarakoro and Bello (2022), and Sam et al. (2022). However, UNEP (2011) indicate that sites remediated and certified by HYPREP still contain elevated contaminants, posing ongoing risks to the local population. Despite remediation interventions, Ihunwo et al. (2021) found that total petroleum hydrocarbons (TPH) were present in Woji Creek, with concentrations ranging from 1.010 to 3.639 mg/L in surface water and 3.162 to 8.758 mg/kg in sediments, highlighting ongoing ecological and potential human health concerns along the creek. Therefore, there is a pressing need to explore alternatives to make meaningful progress in environmental restoration.

Recent studies in the Niger Delta have shed light on the effectiveness of saprobic fungi and various bacterial groups in cleaning up soils contaminated with hydrocarbons, showcasing the capacity for natural pollutant breakdown. Research has particularly pointed out fungi like *Aspergillus oryzae* and *Mucor irregularis* (Asemoloye et al., 2020), for their resilience to used engine oil and their superior hydrocarbon decomposition abilities, highlighted by considerable enzyme activities, notably in laccase. Such discoveries open up valuable opportunities for using local microbial communities to treat oil-contaminated soils.

Together, these findings underline the critical function of microbial populations in breaking down hydrocarbons and the need to fine-tune bioremediation methods to leverage these natural mechanisms effectively. However, applying these research insights to real-world clean-up efforts poses a challenge. It calls for a comprehensive strategy that merges understanding of microbes with sustainable environmental management practices to mitigate the complex pollution problems in the Niger Delta effectively, while these studies were crucial for understanding bioremediation in the Niger Delta, the challenge lies in implementing these findings in actual field applications.

## 6 Conclusions

The profound and persistent environmental crisis in Nigeria's Niger Delta, driven by decades of crude oil contamination, demands an urgent and fundamental re-evaluation of prevailing restoration strategies. Current approaches, particularly the reliance on surface-level bacterial bioremediation, have demonstrably failed, producing a catastrophic performance gap due to a core technical flaw a “depth mismatch” with the deep, aged, and recalcitrant nature of the pollution. This review posits that mycoremediation offers a strategically superior paradigm, one that is uniquely aligned with the specific challenges of the region. Fungi possess a multi-faceted advantage, combining the deep soil penetration of their mycelial networks, a powerful and non-specific enzymatic arsenal for degrading complex hydrocarbons, and the ability to form synergistic consortia with bacteria. Furthermore, mycoremediation exhibits a remarkable ecological congruence with the Niger Delta's environment, as the region's acidic, saline, and mesophilic conditions naturally favor fungal dominance. However, despite these advantages, oil pollution remains a prominent and unresolved issue in the region, underscoring important translational and operational limitations. Field-scale implementation faces challenges including environmental heterogeneity, fluctuating abiotic stressors, competition with indigenous microbial communities, variability in contaminant composition, and uncertainties regarding long-term fungal establishment and stability. Large-scale inoculum production, controlled delivery into contaminated subsurface zones, and sustained monitoring frameworks remain insufficiently developed. Furthermore, limited long-term field validation and integration into regulatory remediation standards have constrained widespread adoption. These limitations highlight that mycoremediation, while promising, is not a standalone remedy but requires rigorous field optimisation, integrated remediation design, and stronger alignment between research, policy, and regulatory institutions.

Moreover, translating this potential into large-scale field success requires a realistic appraisal of implementation constraints. Competition with indigenous microbiota may limit fungal establishment, and fungi generally exhibit slower intrinsic growth rates than many hydrocarbon-degrading bacteria, potentially affecting early-stage degradation kinetics. Large-scale production and field delivery of fungal inoculum remain technically challenging, particularly for specialized hydrocarbonoclastic species that lack established cultivation systems. Many degradative fungi do not have scalable propagation frameworks. Furthermore, some ligninolytic fungi require lignocellulosic amendments to stimulate enzyme production, necessitating soil mixing and limiting fully *in situ* applications, although certain species such as *Trichoderma harzianum* may colonize soils without such amendments.

Mycoremediation should therefore be viewed not as a universal remedy, but as a strategically valuable component within an integrated remediation portfolio. Targeted bioprospecting of indigenous strains, improved inoculum production systems, and robust field-scale validation, fungal-based approaches hold meaningful potential to contribute to ecological restoration, livelihood recovery, and environmental justice in this globally significant oil-producing region.

## Data Availability

All the data supporting this study are included within the article and/or supporting materials.
